# A 20-Year Study of Capsular Polysaccharide Seroepidemiology, Susceptibility Profiles, and Virulence Determinants of Klebsiella pneumoniae from Bacteremia Patients in Taiwan

**DOI:** 10.1128/spectrum.00359-23

**Published:** 2023-05-16

**Authors:** Chun-Chou Tsai, Jung-Chung Lin, Pei-Chen Chen, Esther Yip-Mei Liu, Yu-Kuo Tsai, Chia-Peng Yu, Jia-Je Li, Ching-Hsun Wang, Chang-Phone Fung, Fu-Mei Lin, Feng-Yee Chang, L. Kristopher Siu

**Affiliations:** a Division of Infectious Diseases and Tropical Medicine, Department of Internal Medicine, Tri-Service General Hospital, National Defense Medical Center, Taipei, Taiwan; b National Institute of Infectious Diseases and Vaccinology, National Health Research Institutes, Miaoli, Taiwan; c School of Public Health, National Defense Medical Center, Taipei, Taiwan; d Section of Infectious Diseases, Department of Medicine, Taipei Veterans General Hospital, Taipei, Taiwan; e Graduate Institute of Basic Medical Science, China Medical University, Taichung, Taiwan; f Institute of Microbiology and Immunology, National Defense Medical Center, Taipei, Taiwan; Universidad Andres Bello

**Keywords:** *Klebsiella pneumoniae*, serotyping, resistance, virulence

## Abstract

In this study, we selected bacteremic Klebsiella pneumoniae isolates from the Taiwan Surveillance of Antimicrobial Resistance program. A total of 521 isolates were collected over a period of 2 decades, including 121 from 1998, 197 from 2008, and 203 from 2018. Seroepidemiology showed that the top five capsular polysaccharide types were serotypes K1, K2, K20, K54, and K62, constituting 48.5% of the total isolates, and the respective ratios at each time point have remained similar over the past 2 decades. The antibacterial susceptibility tests showed that K1, K2, K20, and K54 were susceptible to most antibiotics, while K62 was relatively resistant compared to other typeable and nontypeable strains. In addition, six virulence-associated genes, *clbA*, *entB*, *iroN*, *rmpA*, *iutA*, and *iucA*, were predominant in K1 and K2 isolates of K. pneumoniae. In conclusion, serotypes K1, K2, K20, K54, and K62 of K. pneumoniae are the most prevalent serotypes and carry more virulence determinants in bacteremia patients, which may indicate their invasiveness. If further serotype-specific vaccine development is performed, these five serotypes should be included. Since the antibiotic susceptibility profiles were stable over a long duration, empirical treatment may be predicted according to serotype if rapid diagnosis from direct clinical specimens is available, such as PCR or antigen serotyping for serotype K1 and K2.

**IMPORTANCE** This is the first nationwide study to examine the seroepidemiology of Klebsiella pneumoniae using blood culture isolates collected over a period of 20 years. The study found that the prevalence of serotypes remained consistent over the 20-year period, with high-prevalence serotypes associated with invasive types. Nontypeable isolates had fewer virulence determinants than other serotypes. With the exception of serotype K62, the other high-prevalence serotypes were highly susceptible to antibiotics. If rapid diagnosis using direct clinical specimens, such as PCR or antigen serotyping, is available, empirical treatment can be predicted based on serotype, particularly for K1 and K2. The results of this seroepidemiology study could also help the development of future capsule polysaccharide vaccines.

## INTRODUCTION

Klebsiella pneumoniae is an opportunistic pathogen that can cause both community-acquired and nosocomial infectious diseases. For community-acquired infections, liver abscess, pneumonia, and urinary tract infections are common, and complications with meningitis or endophthalmitis are also encountered (1). For nosocomial infections, bacteremia is an important risk factor for mortality (2, 3). The capsular polysaccharides (CPSs) of K. pneumoniae are one of the most important factors in its invasiveness (4). Currently, at least 79 capsular types have been found (5).

Because of the overuse of antibiotics such as β-lactams, antibiotic resistance is emerging and increasing conspicuously, causing most antibiotics, such as broad-spectrum cephalosporins, aztreonam, and monobactam, to no longer be effective for K. pneumoniae infections (6). Hypervirulent K. pneumoniae strains have also frequently been described. These include capsular serotypes K1 and K2 and strains with sequence type 11 (ST11) (7). Multilocus sequence typing (MLST) has been used extensively to study the genetic diversity of K. pneumoniae and to understand the association between specific STs and virulence factors. Several studies have shown that certain K. pneumoniae STs are associated with an increased risk of virulence. For example, ST23, ST65, ST66, and ST86 have been associated with hypervirulent K. pneumoniae strains (8, 9), which are characterized by the presence of a hypermucoviscosity phenotype of serotype K1 or K2 and the ability to cause severe infections in otherwise-healthy individuals. In contrast, other STs, such as ST258 and ST11, are more commonly associated with antibiotic resistance and hospital-associated infections (10). Studies have shown that virulence is highly varied in these hypervirulent strains. Unlike serotypes K1 and K2, which can cause mortality with low concentrations of bacteria, ST11 isolates were reported unable to kill mice even at very high concentrations (7). Thus, serotype is a relatively important identity for determining virulence or for the future use in diagnosing invasive infection (11). In addition to the above virulence types of strains, virulence genes have also been suggested to contribute to the invasiveness of K. pneumoniae, including *rmpA*, *rmpA2*, *clbA*, *entB*, *iroN*, *iutA*, and *iucA*. *rmpA* is the regulator associated with hypermucoviscosity (12). *clbA* is the gene that encodes colibactin. Colibactin synthesis is encoded within the 54-kb genomic island *pks*, representing a total of 19 genes (*clbA* to *clbS*), and is a protein that disrupts DNA and the cell cycle (13). Several siderophores are crucial for Klebsiella pneumoniae. Enterobactin is the product of *entB* and is bound by lipocalin-2, secreted by the host (14). Salmochelin is the C-glucosylated form of enterobactin. The *iroN* gene regulates enterobactin and is common in hypervirulent K. pneumoniae (12, 15). Aerobactin is another siderophore produced by the *iucA* gene (16). Both *iucA* and *iutA* are involved in the synthesis and uptake of aerobactin. *iutA* encodes a protein called aerobactin receptor, which is an outer membrane receptor that allows K. pneumoniae to take up aerobactin (17), while *iucA* encodes a protein called aerobactin synthase which is involved in the biosynthesis of aerobactin. Isolates of K. pneumoniae with these genes may be more virulent than strains without these genes (14).

In Taiwan, K. pneumoniae is one of the most common pathogens of nosocomial and community-acquired infections (18). However, antibiotic resistance has become a problem recognized worldwide due to the long-term use of antibiotics, and treatment is more difficult for clinicians (19). The development of a vaccine for K. pneumoniae is one of the potential control strategies for this infection. There is a successful example of using CPS as a vaccine, similar to the Streptococcus pneumoniae vaccine. Different vaccines, such as the 23-valent pneumococcal CPS vaccine, 7-valent pneumococcal conjugated vaccine, and 13-valent pneumococcal conjugated vaccine, have been developed and used (20). A 24-valent CPS K. pneumoniae vaccine has been developed in Switzerland (21) and confirmed to have immunogenicity and safety (22). Nevertheless, it is not clear exactly which K. pneumoniae CPS should be included in an effective vaccine, owing to as many as 79 serotypes of K. pneumoniae. There are also different common serotypes of K. pneumoniae in various regions globally (23–25). A previous study indicated that the vaccine developed in Switzerland was not suitable for Taiwan because it lacked the predominant serotypes of K1, K54, and K57 (18). The recent serotype distribution of K. pneumoniae has not been investigated, and its epidemiology may have changed over time. In addition, antibiotic resistance may vary for different serotypes of K. pneumoniae. For these reasons, our study investigated isolates of K. pneumoniae in three different longitudinal years, 1998, 2008, and 2018, to determine the seroepidemiology of K. pneumoniae. We hope that these findings will be beneficial for the further development of vaccines in Taiwan, the choice of effective empirical antibiotics, and virulence determinations for further diagnosis of this bacterium.

## RESULTS

### Distribution and serotyping of K. pneumoniae blood culture isolates from 1998 to 2018.

**FIG 1:**
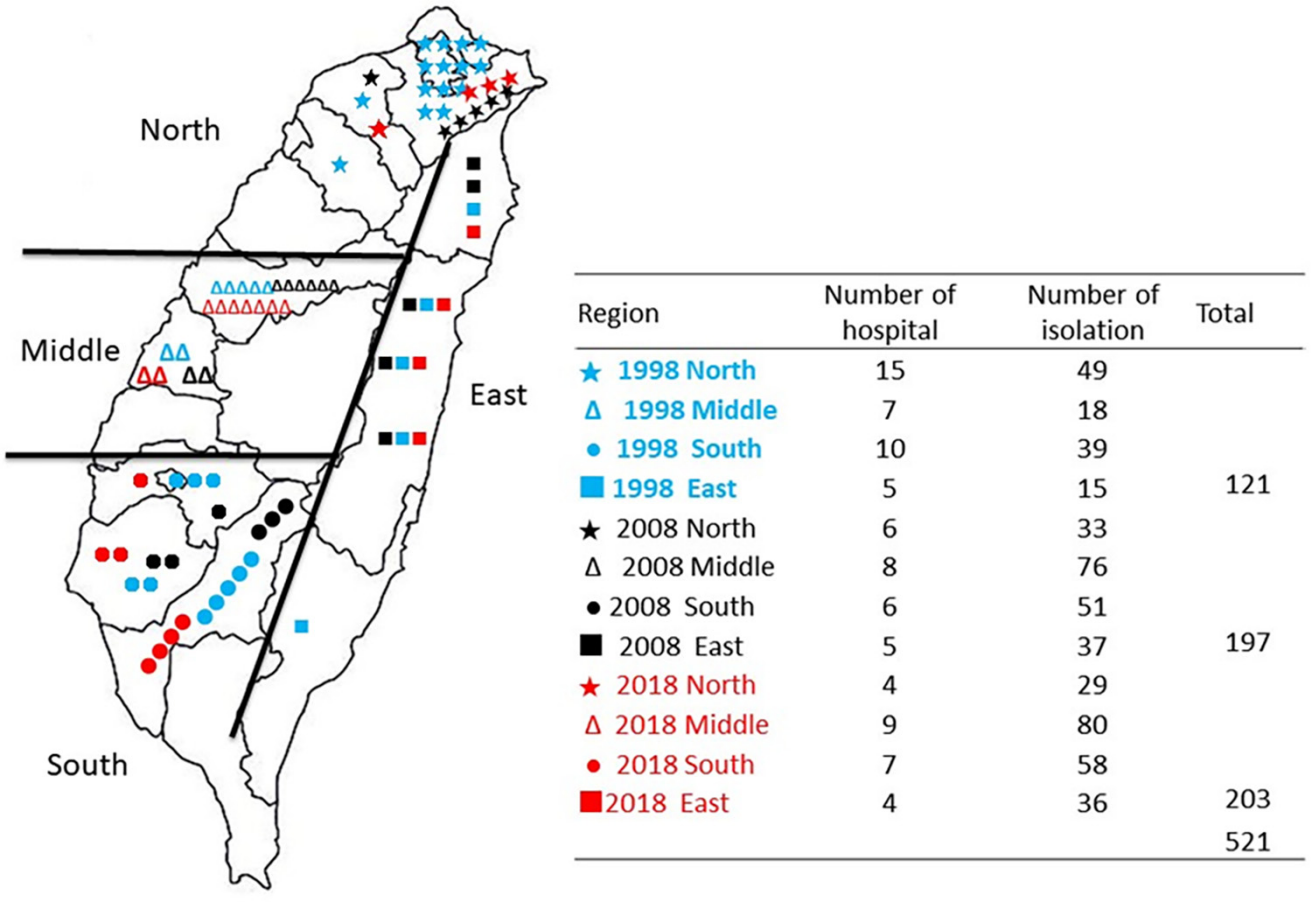
Hospital distribution of blood culture isolates of K. pneumoniae collected from 1998 to 2018 for this study.

Isolates were collected from different regions of Taiwan, and the distribution is presented in Fig. 1. There were 521 isolates collected from hospitals located in Taiwan, including 37 in 1998, 25 in 2008, and 24 in 2018 (Fig. 1). The seroepidemiology of K. pneumoniae blood cultures from 1998 to 2018 was determined (Table 1). Two predominant serotypes, K1 and K2, were identified, which accounted for 16.5% and 15.5% of the total isolates from these 2 decades, respectively. By the nonparametric chi-square test (goodness-of-fit test), serotypes K1 and K2 were significantly more prevalent (*P* < 0.001) in bacteremia than other serotype isolates. Following serotypes K1 and K2, serotypes K20 (6.9%), K54 (5.6%), K62 (4.0%), K5 (3.3%), K64 (2.9%), K16 (2.5%), K24 (2.3%), and K25 (2.3%) were the most common seroprevalent types in the collection, representing 61.8% of the total isolates in this study (Table 1). There were 14.8% of isolates in total that were nontypeable, of which 15.7%, 13.2%, and 15.8% were from 1998, 2008, and 2018, respectively. For other typeable serotypes, the appearance rate was less than 2% in the year of the survey. Serotypes K1, K2, K3, K10, K12, K14, K16, K20, K21, K24, K25, K27, K34, K48, K51, K54, K57, K62, K63, K64, and KN2 were identified in all three consecutive surveys, while K4, K6, K9, K11, K17, K22, K29, K32, K33, K36, K43, K44, K45, K46, K49, K50, K52, K53, K58, K59, K60, K65, K66, K67, K68, K69, K70, K72, K79, K80, K81, K82, and KN1 did not appear in any survey, indicating a nonprevalent infectious type in K. pneumoniae infection (Table 1).

**TABLE 1:** Serotyping of blood-cultured K. pneumoniae isolates collected from 1998 to 2018

Serotype^*a*^	No. (%) of isolates with serotype			
	By yr			Total (%)
	1998 (*n* = 121)	2008 (*n* = 197)	2018 (*n* = 203)	
**K1**	**21 (17.4)**	**34 (17.3)**	**31 (15.3)**	**86 (16.5)** ^*b*^
**K2**	**21 (17.4)**	**31 (15.7)**	**29 (14.3)**	**81 (15.5)** ^*b*^
K3	1 (0.8)	1 (0.5)	1 (0.5)	3 (0.6)
**K5**		**6 (3.0)**	**11 (5.4)**	**17 (3.3)**
K7		3 (1.5)		3 (0.6)
K8		2 (1.0)	2 (1.0)	4 (0.8)
K10	1 (0.8)	1 (0.5)	1 (0.5)	3 (0.6)
K12	1 (0.8)	1 (0.5)	2 (1.0)	4 (0.8)
K13	1 (0.8)		3 (1.5)	4 (0.8)
K14	2 (1.7)	1 (0.5)	1 (0.5)	4 (0.8)
K15		3 (1.5)	7 (3.4)	10 (1.9)
**K16**	**4 (3.3)**	**3 (1.5)**	**6 (3.0)**	**13 (2.5)**
K18		1 (0.5)		1 (0.2)
K19			1 (0.5)	1 (0.2)
**K20**	**8 (6.6)**	**14 (7.1)**	**14 (6.9)**	**36 (6.9)**
K21	2 (1.7)	2 (1.0)	2 (1.0)	6 (1.2)
K23		4 (2.0)	1 (0.5)	5 (1.0)
**K24**	**3 (2.5)**	**4 (2.0)**	**5 (2.5)**	**12 (2.3)**
**K25**	**2 (1.7)**	**5 (2.5)**	**5 (2.5)**	**12 (2.3)**
K26			1 (0.5)	1 (0.2)
K27	3 (2.5)	1 (0.5)	2 (1.0)	6 (1.2)
K28		2 (1.0)	2 (1.0)	4 (0.8)
K30		1 (0.5)		1 (0.2)
K31		3 (1.5)	1 (0.5)	4 (0.8)
K34	1 (0.8)	1 (0.5)	1 (0.5)	3 (0.6)
K35		1 (0.5)	2 (1.0)	3 (0.6)
K37	2 (1.7)	1 (0.5)		3 (0.6)
K38	1 (0.8)	2 (1.0)		3 (0.6)
K39		2 (1.0)	1 (0.5)	3 (0.6)
K40		1 (0.5)		1 (0.2)
K41			1 (0.5)	1 (0.2)
K42	1 (0.8)			1 (0.2)
K47			1 (0.5)	1 (0.2)
K48	1 (0.8)	3 (1.5)	1 (0.5)	5 (1.0)
K51	1 (0.8)	2 (1.0)	2 (1.0)	5 (1.0)
**K54**	**11 (9.1)**	**9 (4.6)**	**9 (4.4)**	**29 (5.6)**
K55	1 (0.8)		3 (1.5)	4 (0.8)
K56			1 (0.5)	1 (0.2)
K57	3 (2.5)	2 (1.0)	3 (1.5)	8 (1.5)
K61	1 (0.8)			1 (0.2)
**K62**	**3 (2.5)**	**12 (6.1)**	**6 (3.0)**	**21 (4.0)**
K63	2 (1.7)	2 (1.0)	2 (1.0)	6 (1.2)
**K64**	**2 (1.7)**	**7 (3.6)**	**6 (3.0)**	**15 (2.9)**
K71		1 (0.5)		1 (0.2)
K74		1 (0.5)		1 (0.2)
KN2	2 (1.7)	1 (0.5)	4 (2.0)	7 (1.3)
Nontypable	19 (15.7)	26 (13.2)	32 (15.8)	77 (14.8)

a The 10 most common serotypes and their prevalence data from 2 decades of K. pneumoniae blood cultures are shown in boldface.

b Serotypes K1 and K2 were significantly more prevalent than other K types (*P* < 0.05).

### Antimicrobial susceptibility of the five most prevalent serotypes in comparison to other serotypes and nontypeable isolates.

According to serotyping, all isolates were intrinsically resistant to ampicillin (data not shown). The 4 most prevalent serotypes, K1, K2, K20, and K54, were highly susceptible to different antibiotics (Table 2). For serotypes K1 and K2, over 91% susceptibility was observed for all tested antibiotics except cefazolin (CFZ), which had 83.7% and 72.8% susceptibility, respectively. Serotypes K1 and K2 were 100% susceptible to imipenem (IMP) and meropenem (MER) over 2 decades. Similar to serotypes K1 and K2, serotypes K20 and K54 were highly susceptible to the tested antibiotics, and >86% of all isolates were susceptible to all tested antibiotics except CFZ, with 77.7% and 68.9% susceptibility observed for K20 and K54, respectively (Table 2). K62 was the fifth most frequently identified serotype among all isolates. Unlike the top 4 serotypes, 42.8% of serotype K62 isolates were susceptible to the β-lactam/β-lactamase inhibitor (AUG). For cephalosporins, 33.3%, 52.3%, 47.6%, 52.3%, and 57.1% of the isolates were susceptible to CFZ, cefoxitin (FOX), cefotaxime (FTX), ceftazidime (CAZ), and cefepime, respectively, indicating that K62 was more resistant to cephalosporins than other serotypes. Over 95% of K62 serotype isolates were still susceptible to IMP and MER. Serotype K62 isolates were also less susceptible to aminoglycosides (GEN and AMK; 52.2% and 71.4%) and quinolone (CIP; 52.2%). Susceptibility tests showed that in addition to the 5 most prevalent serotypes, other serotypes and nontypeable isolates had similar susceptibility profiles. They were more resistant to CFZ (56.5% for other serotypes and 58.8% for nontypeable), in comparison to the most common serotypes of K1, K2, K20, and K54. In contrast, serotypes other than K1, K2, K20, and K54 and nontypeable isolates were more susceptible to CFZ, GEN, and AMK than K62 serotype isolates. Overall, the isolates in this study were highly susceptible to carbapenems and tigecycline, with susceptibility in over 95% (Table 2). There was no significant change in the antibiotic resistance rate from 1998 to 2018 among all isolates.

**TABLE 2:** Susceptibility rates among the 5 most prevalent serotypes and other serotypes collected from 1998 to 2018

Serotype and yr	No. of isolates	% of isolates susceptible to^*a*^:														
		AUG	ATM	CFZ	FOX	CAZ	FTX	FEP	IMP	MER	GEN	AMK	CIP	SXT	TGC	COL
K1																
1998	21	95.2	95.2	85.7	100	100	95	95.2	100	100	95.2	95.2	100	95.2	100	100
2008	34	100	100	85	97	85	100	100	100	100	100	100	97	94.1	100	100
2018	31	93	96	80	96.7	96.7	96.7	96.7	100	100	100	100	100	96.7	100	90.3
Total	86	96.5	97.6	83.7	97.6	93.0	97.6	97.6	100	100	98.8	98.8	98.8	95.3	100	96.5
K2																
1998	21	95.2	95.2	66.6	100	100	95.2	95.2	100	100	80.9	80.9	100	95.2	100	80.9
2008	31	90.3	93.5	93.1	100	87.0	93.5	93.5	100	100	93.5	100	93.5	96.7	96.7	100
2018	29	96.5	96.5	62.0	92.8	96.5	96.5	96.5	100	100	96.5	100	96.5	96.5	100	92.8
Total	81	93.8	95.0	72.8	96.2	93.8	95	95	100	100	91.3	95	96.2	96.2	98.7	91.3
K20																
1998	8	100	100	87.5	87.5	100	100	100	100	100	87.5	87.5	100	75.0	100	87.5
2008	14	78.5	85.7	78.5	78.5	78.5	78.5	85.7	100	100	78.5	78.5	78.5	71.4	100	100
2018	14	85.7	100	71.4	92.8	92.8	92.8	100	92.8	100	92.8	100	92.8	85.7	100	92.8
Total	36	86.1	94.4	77.7	86.1	88.8	88.8	94.4	97.2	100	86.1	88.8	88.8	77.7	100	94.4
K54																
1998	11	81.8	90.9	54.5	72.7	90.9	90.9	90.9	100	100	90.9	90.9	100	72.7	90.9	100
2008	9	88.8	88.8	88.8	88.8	88.8	88.8	88.8	88.8	88.8	100	100	88.8	88.8	100	100
2018	9	100	100	66.6	77.7	77.7	77.7	100	100	100	88.8	100	100	77.7	100	100
Total	29	89.6	93.1	68.9	86.2	86.2	86.2	93.1	96.5	96.5	93.1	96.5	96.5	79.3	96.5	100
K62																
1998	3	66.6	66.6	66.6	66.6	66.6	66.6	66.6	100	100	66.6	66.6	66.6	66.6	100	66.6
2008	12	25	41.6	25	33.3	25	33.3	33.3	91.6	91.6	33.3	58.3	25	50	100	100
2018	6	66.6	83.3	33.3	83.3	83.3	83.3	100	100	100	83.3	100	100	66.6	100	66.6
Total	21	42.8	57.1	33.3	52.3	47.6	52.3	57.1	95.2	95.2	52.3	71.4	52.3	57.1	100	85.7
Other K types																
1998	38	81.5	89.4	71	81.5	89.4	81.5	84.2	100	100	76.3	89.4	94.7	94.7	92.1	86.8
2008	71	71.8	77.4	60.5	76	78.8	76	85.9	97.1	97.1	71.8	83	77.4	63.3	100	100
2018	82	98.7	81.7	46.3	62.1	64.6	67	87.8	98.7	98.7	69.5	98.7	78	59.7	90.2	91.4
Total	191	85.3	81.6	56.5	71.2	74.8	73.2	86.3	98.4	98.4	71.7	91.9	81.1	62.8	94.2	93.7
Nontypeable																
1998	19	89.4	89.4	47.3	68.4	89.4	84.2	94.7	100	100	68.4	78.9	84.2	68.4	94.7	84.2
2008	26	80.7	100	76.9	84.6	96.1	92.3	100	96.1	100	84.6	100	84.6	73.0	100	100
2018	30	66.6	90	50	70	66.6	60	93.3	100	96.6	70	100	90	56.6	100	100
Total	75	77.3	93.3	58.6	74.6	82.6	77.3	966	98.6	98.6	74.6	94.6	86.6	65.3	98.6	96

a AUG, amoxicillin-clavulanic acid; ATM, aztreonam; CFZ, cefazolin; FOX, cefoxitin; FRX, cefuroxime; CAZ, ceftazidime; FTX, cefotaxime; FEP, cefepime; IMP, imipenem; TGC, tigecycline; GEN, gentamicin; AMK, amikacin; CIP, ciprofloxacin; SXT, trimethoprim-sulfamethoxazole; MER, meropenem; COL, colistin.

### Distribution of virulence-associated determinants among different serotypes of isolates.

According to the serotype distribution, the prevalence of virulence-associated determinants is listed in Table 3. The virulence-associated determinants varied in prevalence among different serotypes of isolates. With reference to the serotypes, there was no extreme difference in the gene prevalence rate among isolates from 1998, 2008, and 2018, indicating gene stability in different serotype isolates. Of all serotypes and nontypeable isolates, 100% carried the *entB* gene from 1998 to 2018, indicating its indistinguishability in virulence in K. pneumoniae. For *iroN*, the prevalence rate declined from high-prevalence K serotypes to low-prevalence K serotypes, with 0 prevalence for nontypeable isolates. Over 91% of serotypes K1 and K2 carried *iroN*, followed by 80% of K20, 68% of K54, 38% of K62, and 15% of other K types. No detection was found in nontypeable isolates (Table 3). Since *iucA* and *iutA* are functional genes for aerobactin expression, they appeared together. Only very few isolates contained either *iucA* or *iutA*. A similar phenomenon to *iroN* was also observed in *iucA* and *iutA*; the prevalence was highest in serotypes K1 and K2, followed by K20, K54, and K62. A low prevalence of *iucA*, 17%, and *iutA*, 18%, was observed for K types other than the 5 most prevalent serotypes. Almost zero identification was found for *iucA* (1%), and *iutA* was undetected in the nontypeable isolates (Table 3). Similar to *iroN*, *iucA*, and *iutA*, the prevalence of *rmpA* also decreased, from 90% and 91% of serotypes K1 and K2, to 80% for K20, 65% for K54, 38% for K62, and 15% for other K types. No *rmpA* was found in nontypeable isolates. The prevalence of *rmpA2* was similar to that of *rmpA* but slightly less than that for *rmpA* (Table 3). Also, the prevalence of *rmpA2* decreased from serotype K1 (87%) and K2 (85%) to 83% for K20, 65% for K54, and 52% for K62. Only 1 nontypeable isolate (1%) was carrying *rmpA2*. These four genes, *iroN*, *rmpA*, *iucA*, and *iutA*, had a direct correlation with the serotype prevalence rate. *clbA* was distributed more frequently in serotypes K1, K2, K20, and K62 and was almost undetected in other isolates, including the highly prevalent serotype K54.

**TABLE 3:** Virulence determinants among different serotypes of bacteremia K. pneumoniae isolates

Serotype and yr	No. of isolates	No. (%) of isolates with virulence determinant						
		*clbA* (%)	*entB* (%)	*iroN* (%)	*iucA* (%)	*iutA* (%)	*rmpA* (%)	*rmpA2* (%)
K1								
1998	21	12 (57)	20 (95)	21 (100)	20 (95)	20 (95)	18 (85)	16 (76)
2008	34	23 (68)	34 (100)	29 (85)	31 (91)	33 (97)	31 (91)	30 (88)
2018	31	23 (74)	31 (100)	29 (93)	30 (96)	29 (93)	29 (93)	29 (93)
Total	86	58 (67)	85 (98)	79 (91)	81 (94)	82 (95)	78 (90)	75 (87)
K2								
1998	21	3 (14)	21 (100)	21 (100)	21 (100)	20 (95)	21 (100)	17 (80)
2008	31	10 (32)	31 (100)	28 (90)	28 (90)	28 (90)	28 (90)	27 (87)
2018	29	12 (41)	28 (96)	25 (86)	25 (86)	24 (82)	25 (86)	25 (86)
Total	81	25 (30)	80 (98)	74 (91)	74 (91)	71 (88)	74 (91)	69 (85)
K20								
1998	8	3 (37)	8 (100)	8 (100)	8 (100)	8 (72)	8 (100)	7 (87)
2008	14	4 (28)	14 (100)	9 (64)	10 (71)	6 (66)	9 (64)	11 (78)
2018	14	7 (50)	14 (100)	12 (85)	11 (78)	5 (55)	12 (85)	12 (85)
Total	36	14 (38)	36 (100)	29 (80)	29 (80)	19 (65)	29 (80)	30 (83)
K54								
1998	11	0	11 (100)	8 (72)	8 (72)	2 (66)	7 (63)	8 (72)
2008	9	0	9 (100)	6 (66)	6 (66)	6 (66)	6 (66)	6 (66)
2018	9	1 (11)	9 (100)	6 (66)	6 (66)	4 (66)	6 (66)	5 (55)
Total	29	1 (3)	29 (100)	20 (68)	20 (68)	12 (57)	19 (65)	19 (65)
K62								
1998	3	2 (66)	3 (100)	2 (66)	2 (66)	2 (66)	2 (66)	2 (66)
2008	12	2 (16)	12 (100)	3 (25)	3 (25)	6 (50)	3 (25)	6 (50)
2018	6	3 (50)	6 (100)	3 (50)	3 (50)	4 (66)	3 (50)	3 (50)
Total	21	7 (33)	21 (100)	8 (38)	8 (38)	12 (57)	8 (38)	11 (52)
Other K types								
1998	38	0	38 (100)	6 (15)	6 (15)	9 (23)	6 (15)	13 (34)
2008	71	0	70 (98)	5 (7)	10 (14)	8 (11)	8 (11)	5 (7)
2018	82	2 (1)	81 (100)	18 (21)	17 (20)	19 (23)	15 (18)	14 (17)
Total	191	2 (1)	189 (98)	29 (15)	33 (17)	36 (18)	29 (15)	32 (16)
Nontypeable								
1998	19	0	19 (100)	0	0	0	0	0
2008	26	0	26 (100)	0	0	0	0	0
2018	30	1 (3)	30 (100)	0	1 (3)	0	0	1 (3)
Total	75	1 (1)	75 (100)	0	1 (3)	0	0	1 (1)

## DISCUSSION

In this study, a long-duration epidemiological study of K. pneumoniae was analyzed. Associations among serotypes, antimicrobial susceptibility, and virulence-associated determinants were evaluated. These data can help to delineate the changes in bacterial species after long-term environmental and clinical selection, including parallel changes in the practice of antibiotic use and new antibiotic development. Our data have shown that the predominant serotypes of blood culture K. pneumoniae in the 2 decades from 1998 to 2018 were almost the same, indicating the possibility of serotype-associated invasiveness. Serotypes K1 (16.5%), K2 (15.5%), K20 (6.9%), K54 (5.6%), and K62 (4.0%) were the five most prevalent serotypes in our study. Although there is a slight difference in the serotype prevalence compared to a previous study conducted in 1993 to 1997 (18), the isolates were from a single hospital and from different clinical specimens. Serotypes K20 and K62 were not listed in the five most predominant serotypes in the single-hospital study. However, serotypes K1, K2, and K54 were compatible in both studies; these serotypes were listed in the five most common serotypes, and serotypes K1 and K2 were the most prevalent serotypes among blood culture isolates (18). In one study with isolates from North America and Europe, serotype prevalence differed from those in our study (24). The predominant serotypes of 703 isolates from blood were K2 (8.9%), K21 (7.8%), K55 (4.8%), K53 (2.8%), K25 (2.8%), and K68 (2.5%). Another study from Australia showed that the predominant serotypes of 293 isolates from different sources were K54 (17.1%), K28 (4.1%), K17 (3.1%), and K1 (2.0%) (25). In China, the predominant serotypes of 348 isolates from different sources were K14 (16.4%), K64 (16.4%), K1 (14.6%), K2 (8.0%), and K57 (5.5%) (26). Those studies and our present data have shown that serotype prevalence varies from region to region. However, unlike our study, the isolates were collected from different sites. Whether the collection site differences contributed to the difference in serotype prevalence needs to be further studied. The reason for the serotype difference could perhaps be confirmed by further study from our Taiwan Surveillance of Antimicrobial Resistance (TSAR) program by using different sites of isolation other than blood culture isolates.

For antimicrobial susceptibility, the 4 most prevalent serotypes of isolates and nontypeable isolates showed a relatively stable susceptibility to all tested antibiotics. K62, which belongs to multilocus sequence type ST11, was the most resistant serotype in this study. Other K types or nontypeable isolates showed relatively more resistance to most antibiotics except to carbapenems, IMP and MER.

Carbapenems are still the most effective antibiotics against all isolates. The 4 most prevalent serotypes were 100% sensitive. The rest of the other isolates had >95% susceptibility to carbapenems. Overall, serotypes K1, K2, K20, and K54 and nontypeable isolates were relatively susceptible to most antibiotics, except for CFZ and FOX. Thus, using third-generation cephalosporins in combination with an aminoglycoside could be a safe empirical treatment for K. pneumoniae when the susceptibility profile is not available in a timely manner. Recently, rapid serotype K1 and K2 diagnostic kits have been developed and used for clinical applications in direct clinical specimens (27, 28). They have shown a high predictive value for K. pneumoniae infection. Combining rapid diagnosis with local epidemiology data may give physicians a crude idea of the use of empirical treatment until the susceptibility data from the clinical laboratory confirm whether changing the treatment is necessary. Currently, serotypes K1 and K2 are the two most prevalent and invasive serotypes in community-acquired infections, and susceptibility to these two serotypes has been stable in the past 20 years. Rapid diagnosis may not only help in disease identification but also provide hints for empirical treatment.

For the correlation between serotypes and virulence determinants, we observed that 100% of isolates carried *entB*. This gene seems to be intrinsic in K. pneumoniae and may not contribute to the virulence differences in K. pneumoniae. In future studies, this gene could be excluded, since it cannot be used as a marker to differentiate virulence or disease severity. One intriguing observation is that the nontypeable isolates contained almost no tested virulence determinants except intrinsic *entB*. In addition, serotypes other than the 5 most prevalent serotypes also had a relatively low prevalence of the tested virulence determinants, which could indicate that carrying more virulence factors provides more opportunities to cause diseases due to their invasiveness. Unfortunately, there are no clinical details of the patients’ immune status for analysis. An invasive syndrome of K. pneumoniae has been previously suggested, where diabetic patients infected with serotype K1 or K2 are at high risk for complications (29). In addition to antibiotic treatment, information on virulence markers and patients' immune status can assist physicians in anticipating the potential complications of endophthalmitis, meningitis, or necrotizing fasciitis. If clinical data are available, the relationship between virulence markers and the occurrence of complications could be analyzed (30). Previously, a 24-variant K. pneumoniae CPS vaccine was developed in Switzerland, but it did not include the predominant capsules of K1, K20, and K54 found in this study. In the future, development of K. pneumoniae vaccines, as with Streptococcus pneumoniae, the serotype distributions from various regions should be obtained to increase the vaccine’s suitability for different regions. Recently, a conjugated K. pneumoniae vaccine was also developed for serotypes K1 and K2 (31). This type of vaccine has been shown to provide effective protection and induce a sufficient immune response even in children with nonmature immunity. If there is further development of a K. pneumoniae-conjugated vaccine in Taiwan, including K20, K54, and K62 and the globally spreading carbapenem-resistant serotypes may be suitable for a high possibility of coverage.

In conclusion, serotypes K1, K2, K20, K54, and K62 were the top 5 prevalent serotypes in blood culture K. pneumoniae in Taiwan, and the prevalence was stable for 20 years. These serotypes should be included if a CPS vaccine is to be developed. The virulence-associated gene *entB* was present in all K. pneumoniae isolates and is possibly intrinsic in K. pneumoniae. The detection of *entB* may be unnecessary in the future. Virulence genes, *iroN*, *rmpA*, *iucA*, and *iutA* are almost undetected from nontypeable isolates. There was an association between antimicrobial susceptibility and serotypes. Serotypes K1, K2, K20, and K54 were highly susceptible to most of the antibiotics, and serotype K62 was more resistant than other K. pneumoniae serotypes when a susceptibility test result was not available. The choice of effective empirical antibiotic treatment could be considered if rapid diagnosis of serotype tests is available.

## MATERIALS AND METHODS

### Collection of isolates.

Taiwan Surveillance of Antimicrobial Resistance (TSAR) is a surveillance program that is run by the National Health Research Institutes. Every other year, bacterial isolates are collected from hospitals that participate in the TSAR program (see the Appendix). The program was initiated in 1998, and isolates have been continually collected up to the present. In this study, we selected 2 months of nonrepeated isolates of blood cultures K. pneumoniae in 1998, 2008, and 2018 from TSAR. There were 121 isolates, 197 isolates, and 203 isolates collected from 1998, 2008, and 2018, respectively (Fig. 1).

### Serotyping by rapid antigen tests, PCR typing, and sequencing.

The isolates were serotyped by rapid cassette to classify the K1, K2, and non-K1/K2 groups (32). We used serotype-specific primer sets to perform multiplex PCR for serotyping. If nontypeable isolates were observed from the first two serotyping tests, then *wzi*, *wza*, and *wzc* gene sequencing was performed to confirm the serotype. PCR and sequencing of these genes were performed according to methods reported previously (33–36) and summarized in Table S1 in the supplemental material.

### Virulence-associated gene detection and antibiotic susceptibility testing.

The genes *iroN*, *clbA*, *entB*, *rmpA*, *rmpA2*, *iutA*, and *iucA* were detected to study the virulence of K. pneumoniae. The primer set is listed in Table S2 (7). The *Taq* polymerase we used for *entB* was *amaR* (One PCR HotStar, Taiwan), and for the other virulence factors, *iroN*, *clbA*, *rmpA*, *rmpA2*, *iutA*, and *iucA*, we used a Q-Amp 2× ScreeningFire *Taq* master mix (Taiwan).

### Antimicrobial susceptibility testing.

We tested the susceptibility of isolates to amikacin, gentamicin, amoxicillin-clavulanate, piperacillin-tazobactam, ampicillin, cefazolin, ceftazidime, cefepime, cefoxitin, cefuroxime, cefotaxime, imipenem, meropenem, ciprofloxacin, trimethoprim-sulfamethoxazole, and aztreonam by means of the broth microdilution method. Susceptibility of isolates to tigecycline and colistin was determined by Etest. The MICs were interpreted following the standards of the Clinical and Laboratory Standards Institute (37), except for tigecycline and colistin. The Food and Drug Administration breakpoint was used for tigecycline, and for colistin, we used the European Committee on Antimicrobial Susceptibility Testing breakpoint (38).

## Appendix

The hospitals that participated in the TSAR program for collection of isolates were as follows:

Hua-Lien Hospital, Hualien Tzu Chi Hospital, Mackay Memorial Hospital, Mennonite Christian Hospital, Chang-Hwa Christian Hospital, Cheng Ching Hospital, China Medical University Hospital, Chun Shan Medical University Hospital, Kuan-Tien General Hospital, Shalu Tung’s Memorial Hospital, Show Chwan Memorial Hospital, Taichung Hospital, Taichung Veterans General Hospital, Zen Ai General Hospital, Cathay General Hospital, Cheng Hsin General Hospital, Cheng Hsin General Hospital, Far Eastern Memorial Hospital, Keelung Chang Gung Memorial Hospital, Koo Foundation Sun Yat-Sen Cancer Center, Linkou Chang Gung Memorial Hospital, Lotung Poh-Ai Hospital, National Taiwan University Hospital, Saint Mary’s Hospital, Taipei City Hospital Heping Fuyou Branch, Taipei City Hospital Zhongxiao Branch, Taipei Hospital MOH, Taipei Medical University Hospital, Taiwan Adventist Hospital, Tao Yuan General Hospital, Tri-Service General Hospital, Chi Mei Medical Center, Chia-Yi Christian Hospital, God’s Help Hospital, Kaosiung Armed Force General Hospital, Kaoshiung Chang Gung Memorial Hospital, Kaosiung Medical University Chung Ho Hospital, Kaosiung Veterans General Hospital, National Cheng Kung University Hospital, The Presbyterian Church Sin-Lau Hospital, St. Martin De Porres Hospital, Tainan Municipal Hospital, and Yuan’s General Hospital.
