# Supplementary material for: A 20-Year Study of Capsular Polysaccharide Seroepidemiology, Susceptibility Profiles, and Virulence Determinants of Klebsiella pneumoniae from Bacteremia Patients in Taiwan
Source: Microbiol Spectr. 2023 May 16;11(3):e00359-23. doi: 10.1128/spectrum.00359-23 (PMC10269490; doi:10.1128/spectrum.00359-23)
Supplement: Supplemental file 1 — Supplemental material. Download spectrum.00359-23-s0001.docx, DOCX file, 0.07 MB [file spectrum.00359-23-s0001.docx]

Supplementary Table 1. Primer sets used in this study for the identification of *K. pneumoniae* serotypes

| Serotype | Primer name | Sequence | Expected amplicon | Accession no. | Reference |
| --- | --- | --- | --- | --- | --- |
| K1 | wzyK1F  wzyK1R | 5′-GGTGCTCTTTACATCATTGC-3′  5′-GCAATGGCCATTTGCGTTAG-3′ | 1283 bp | AB924547 | (29) |
| K2 | wzyK2F  wzyK2R | 5'-GACCCGATATTCATACTTGACAGAG-3'  5'-CCTGAAGTAAAATCGTAAATAGATGGC-3' | 641 bp | AB371296 | (29) |
| K3 | WzyK3F  WzyK3R | 5'-TAGGCAATTGACTTTAGGTG-3'  5'-AGTGAATCAGCCTTCACCT-3' | 549 bp | FQ311478 | (39) |
| K4 | K4wzcF  K4wzcR | 5'-CATCATCGGGATTACAGC-3'  5'-GGCAGGCACATTGAGACG-3' | 337 bp | AB924548 | This study |
| K5 | K5wzxF  K5wzxR | 5'-TGGTAGTGATGCTCGCGA-3'  5'-CCTGAACCCACCCCAATC-3' | 280 bp | AB371292 | (29) |
| K5 | K5wzcF  K5wzcR | 5'-CTTAGTTCTCGTTCGTG-3'  5'-AGCCCATTATCATTACTC-3' | 426 bp | AB371292 | This study |
| K6 | K6wzcF  K6wzcR | 5'-ATGACTGAAAGAACCAAGCA-3'  5'-TGATCGTATTCTAGCCGTG-3' | 350 bp | AB924549 | This study |
| K7 | K7wzyF  K7wzyR | 5'-TCGCAAGAGTTAGTTATT-3'  5'-CCTATCAGAGGCAAAAG-3' | 960 bp | AB924550 | This study |
| K8 | K8wzyF  K8wzyR | 5'-AGGGGATAGCGTAGGTA-3'  5'-TAGAATGCCCAAACTGT-3' | 933 bp | AB924551 | This study |
| K9 | K9wzyF  K9wzyR | 5'-ATGGTGATTATGAATGAAG-3'  5'-ACACAATGAAAACATTGCC-3' | 1283 bp | AB371293 | (28) |
| K10 | K10wzyF  K10wzyR | 5'-GTATTATTCAGTATGTCGCT-3'  5'-GCAAAAGTAGATGAGGTTA-3' | 644 bp | AB924552 | This study |
| K11 | K11wzcF  K11wzcR | 5'-TTGATGAAGCCGTTACAC-3'  5'-ACCAGCATTCGGACTAG-3' | 393 bp | AB924553 | This study |
| K12 | K12wzyF  K12wzyR | 5'-TCGTCTTTACTACCTCCG-3'  5'-AATACCCCTGACACTGC-3' | 576 bp | AB924554 | This study |
| K13 | K13wzyF  K13wzyR | 5'-TTATTTGCTTACCTTGC-3'  5'-TATGTCTGAACCTCACTAA-3' | 768 bp | AB924555 | This study |
| K14 | K14wzyF  K14wzyR | 5'-GACTCTGAATAAAAGAACAC-3'  5'-CTCAATAAATCTGTTCTGAAG-3' | 1209 bp | AB371294 | (28) |
| K15 | K15wzyF  K15wzyR | 5'-TACCCATAGCTATATGCGGC-3'  5'-GGGAAAGTTGCAGCATATTC-3' | 800 bp | AB924556 | (28) |
| K16 | K16wzyF  K16wzyR | 5'-ATGGTACCGTTGGGGTTATC-3'  5'- TAATCAACAATGTCGTAGCG-3' | 742 bp | AB742228 | (28) |
| K17 | K17wzyF  K17wzyR | 5'-TAATGGGGACGAATCAG-3'  5'-CGCTAACGAATAGACCA-3' | 500 bp | AB924557 | This study |
| K18 | K18wzyF  K18wzyR | 5'-GAGGATGTGACAAAGCC-3'  5'-AACCCTAACCCTAAACC-3' | 611 bp | AB924558 | This study |
| K19 | K19wzyF  K19wzyR | 5'-GTTATGTAATAGGCTTTTC-3'  5'-TATTCGCACCATAGTTC-3' | 654 bp | AB924559 | This study |
| K20 | K20wzyF  K20wzyR | 5'-CGGTGCTACAGTGCATCATT-3'  5'-GTTATACGATGCTCAGTCGC-3' | 741 bp | AB371289 | (39) |
| K21 | K21wzyF  K21wzyR | 5'-TTCTTAGTTTGTTGGTGT-3'  5'-TTACTTGCTCTGCTGTT-3' | 577 bp | AB924560 | This study |
| K22 | K22wzcF  K22wzcR | 5'-ATTCTGGGATGCTTACTC-3'  5'-ACCTATCGCACTTGACTT-3' | 539 bp | AB819893 | This study |
| K23 | K23wzyF  K23wzyR | 5'-TGAACTATTCGGTTTACG-3'  5'-CTATTTGCTCCAACACTA-3' | 951 bp | AB924561 | This study |
| K24 | K24wzcF  K24wzcR | 5'-TGTTGAGCGACAAGCAGC-3'  5'-TCAGCAGGATTAGGAGGC-3' | 1074 bp | AB924562 | This study |
| K25 | K25wzyF  K25wzyR | 5'-GTTTACTCTGCTCTATTTAC-3'  5'-TTCAACCTTCTCCTACT-3' | 902 bp | AB924563 | This study |
| K26 | K26wzyF  K26wzyR | 5'-TAGTCTGGGTCTATCTGA-3'  5'-ACTGTGACAACAAATGG-3' | 1101 bp | AB924564 | This study |
| K27 | K27wzcF  K27wzcR | 5'-TCAGCCTCAATCAGCACC-3'  5'-TTCAAACCTGGCAACAAG-3' | 1250 bp | AB924565 | This study |
| K28 | K28wzyF  K28wzyR | 5'-GCGTGACACTTCGTTAT-3'  5'-TCCACCGATACCACCAT-3' | 683 bp | AB924566 | This study |
| K29 | K29wzxF  K29wzxR | 5'-AGAACAGGCACTATTACG-3'  5'-GCTTACAAGATACCGAAA-3' | 763 bp | AB924567 | This study |
| K30 | K30wzyF  K30wzyR | 5'-CAACTAATGTTTCCCTCG-3'  5'-TAGATTGCATAACCCTCA-3' | 738 bp | AB924568 | This study |
| K31 | K31wzyF  K31wzyR | 5'-TAACAATCACTTTATCGCTG-3'  5'-CAAAGCTGACATACAAATGA-3' | 442 bp | AB924569 | This study |
| K32 | K32wzyF  K32wzyR | 5'-ATGAGGGTTATTAGCAATCT-3'  5'-CAGATTCACCCGTTTAAATA-3' | 564 bp | AB924570 | This study |
| K33 | K33wzyF  K33wzyR | 5'-TATTGCCCATAACGAGC-3'  5'-GATAACCGATGAGCGAC-3' | 1090 bp | AB924571 | This study |
| K34 | K34wzyF  K34wzyR | 5'-AGATGGCTTTACAATGC-3'  5'-CAGGATGGAAAACAGTG-3' | 582 bp | AB924572 | This study |
| K35 | K35wzcF  K35wzcR | 5'-CCTGGTGCTGTATTTGAT-3'  5'-CCTGATTCTACATCCCTAC-3' | 560 bp | AB924573 | This study |
| K36 | K36wzyF  K36wzyR | 5'-GCATAATGACACAGAGAGGA-3'  5'-AATGGATTAGTGCCGCTATC-3' | 351 bp | AB924574 | This study |
| K37 | K37wzyF  K37wzyR | 5'-CCTTAGCAATACCAACT-3'  5'-CAATAACAAACCTCCAC-3' | 804 bp | AB924575 | This study |
| K38 | K38wzyF  K38wzyR | 5'-GATGAGGGCATAAGTAA-3'  5'-AATGAAATAGCGATAAGA-3' | 527 bp | AB924576 | This study |
| K39 | K39wzyF K39wzyR | 5'-ATGACCAATGACTTACAAAG-3'  5'-GAATTCCGTTCCAGCCCAC-3' | 1100 bp | AB742230 | (28) |
| K40 | K40wzyF  K40wzyR | 5'-GAGAACAACTACGGCTTT-3'  5'-CACCACAATAAAACTAACC-3' | 503 bp | AB924577 | This study |
| K41 | K41wzyF  K41wzyR | 5'-TCTTTGCTACATCAGGA-3'  5'-TAATAACCCACAGGACA-3' | 879 bp | AB924578 | This study |
| K42 | K42wzcF  K42wzcR | 5'-TACCCGATAGCCAACCAC-3'  5'-AGACAGCACGCCCAGATT-3' | 924 bp | AB924579 | This study |
| K43 | K43wzyF  K43wzyR | 5'-CGACTTTGGTTTCCTTC-3'  5'-CTTGACCTTTCCTTTCC-3' | 532 bp | AB924580 | This study |
| K44 | K44wzcF  K44wzcR | 5'-CAGACAGCCAACCACAAT-3'  5'-AATACACGCAGAAGCACC-3' | 1108 bp | AB924581 | This study |
| K45 | K45wzyF  K45wzyR | 5'-TGTATGCTCTTAGTCTTTC-3'  5'-CTCAGTAATCCTTTTCG-3' | 842 bp | AB924582 | This study |
| K46 | K46wzyF  K46wzyR | 5'-GACAAAACCTGTATCACTG-3'  5'-AAAACCTATTAGCACGC-3' | 988 bp | AB924583 | This study |
| K47 | K47wzyF2  K47wzyR2 | 5'-TACACAAGGGAAAATTAACATCACC-3'  5'-CCCTGGGATAGGAAAATTTCAATAT-3' | 961 bp | AB924584 | This study |
| K48 | K48wzcF  K48wzcR | 5'-GGGACGAGCGGTTTACAT-3'  5'-TATTATTGCGGCATCAGT-3' | 823 bp | LT174559 | This study |
| K49 | K49wzyF  K49wzyR | 5'-GTCGAGCTACTACCGTTAGG-3'  5'-CACGTATAGCTTTCATCAGC-3' | 733 bp | AB924586 | This study |
| K50 | K50glyF  K50glyR | 5'-CCAATGATAATACTGCGCAG-3'  5'-CAACCCGATCATATCATCTC-3' | 616 bp | AB924587 | (28) |
| K51 | K51wzyF  K51wzyR | 5'-TATGGGAGTTGAAGTAGA-3'  5'-GTAAAGCGGAGATAAGTG-3' | 379 bp | AB924588 | This study |
| K52 | K52F  K52R | 5'-GACAAAGTCACCAGCAA-3'  5'-CCAAATCGCAGTAACAC-3' | 500 bp | AB924589 | This study |
| K53 | K53wzyF  K53wzyR | 5'-TGTTCGTTTCATTTGAC-3'  5'-AATACCAGGAGAAGATAGTA-3' | 395 bp | AB924590 | This study |
| K54 | K54wzyF  K54wzyR | 5'-TTACCTCAGAGCGTTGCATTG-3'  5'-TTAGGTATGACAATTGAGCTC-3' | 953 bp | AB924591 | (28) |
| K55 | K55wzcF  K55wzcR | 5'-TATTGATGCCGATTTACG-3'  5'-ATTGGAACGCCACTTTGT-3' | 390 bp | AB924592 | This study |
| K56 | K56wzyF  K56wzyR | 5'-TTCGGAATGGTTGAGTA-3'  5'-AACAAGTCGTTTAGATAGG-3' | 891 bp | AB924593 | This study |
| K57 | K57wzyF  K57wzyR | 5'-CTCAGGGCTAGAAGTGTCAT-3'  5'-CACTAACCCAGAAAGTCGAG-3' | 1037 bp | AB924594 | (39) |
| K58 | K58wzcF  K58wzcR | 5'-GACAAATAAAGGCGGTAA-3'  5'-CTTCAAATAGAGCGACCA-3' | 560 bp | LT174567 | This study |
| K59 | K59wzcF  K59wzcR | 5'-CTAAAGGGAAACGGAAGA-3'  5'-CAAGTATTGGCGGTGTAT-3' | 498 bp | AB924596 | This study |
| K60 | K60wzyF  K60wzyR | 5'-TGTACTAGTTAGTGTGCCCG-3'  5'-GCATCTACGGAATATCTTGA-3' | 500 bp | AB924597 | This study |
| K61 | K61wzyF  K61wzyR | 5'-GCTAATGCTGCTGGTTGT-3'  5'-GTTATGCGGTGGGTAAAT-3' | 447 bp | AB924598 | This study |
| K62 | K62wzyF  K62wzyR | 5'-ATGTCAGTGATTATTTCAGG-3'  5'-AGAGTATGTCATCACGCACG-3' | 1050 bp | AB371295 | (28) |
| K63 | K63wzcF  K63wzcR | 5'-TGTTTGGAAAGGGATGGG-3'  5'-ATGAAGGCTGGTGCGTAA-3' | 1202 bp | AB924599 | This study |
| K64 | K64wzyFnew  K64wzyRnew | 5'-CTTTTAGGGCTACGGCACC-3'  5'-CCGCGCGCAGGAACATTAG-3' | 700 bp | AB924600 | (40) |
| K65 | K65wzyF  K65wzyR | 5'-GGATTACTAACTCAAACAC-3'  5'-ATAAGAAAGAAACGACTAG-3' | 521 bp | AB924601 | This study |
| K66 | K66wzyF  K66wzyR | 5'-TTGTTTTGCGATTCTTA-3'  5'-GAGTCATTAGCCCAGTC-3' | 563 bp | AB924602 | This study |
| K67 | K67wzyF  K67wzyR | 5'-ATCTTCAGAACATGATCCGG-3'  5'-TCAGCAATACCAAAGCAATT-3' | 902 bp | AB924603 | This study |
| K68 | K68wzyF  K68wzyR | 5'-GAAGGGTGTTCGTAGTA-3'  5'-CAACTCAGGAAGAATAAC-3' | 835 bp | AB924604 | This study |
| K69 | K69wzcF  K69wzcR | 5'-TGAACAATGAAGAGGGAA-3'  5'-ACCAAGCCAATAAGAACA-3' | 868 bp | AB720689 | This study |
| K70 | K70wzyF  K70wzyR | 5'-TAATATTTATTGCTGCGTGC-3'  5'-CAGCATTGCAATAAGTATGA-3' | 401 bp | AB924606 | This study |
| K71 | K71wzxF  K71wzxR | 5'-TTTCTCGGCAGGTGTCT-3'  5'-TCGGTCAAAAGTGTCGC-3' | 540 bp | AB924607 | This study |
| K72 | K72wzcF  K72wzcR | 5'-TTTAGGTCGTCTTATTGG-3'  5'-ACTTCGCACTTCAGGTAG-3' | 787 bp | AB924608 | This study |
| K74 | K74wzyF  K74wzyR | 5'-AACACTAGAAACTATACATCCGT-3'  5'-AAAGCCTCCCAATCCAG-3' | 996 bp | AB924609 | This study |
| K79 | K79wzcF  K79wzcR | 5'-CAGGAGATAAAGCGGGTA-3'  5'-CTGGTTTGGGTTGGGTCA-3' | 873 bp | AB924610 | This study |
| K80 | K80wzyF  K80wzyR | 5'-ATTTTAGCATTTACCATCC-3'  5'-ATCCCATCCATTCACAA-3' | 813 bp | AB924611 | This study |
| K81 | K81wzyF  K81wzyR | 5'-TCTTATGCGGTTGTCTGT-3'  5'-CTGATGTAAGCGTGAATG-3' | 374 bp | AB924612 | This study |
| K82 | K82wzyF  K82wzyR | 5'-ATGTTACCCTATGTCTTAGTTC-3'  5'-ACACCACTAAAGAAGAATGT-3' | 404 bp | AB924613 | This study |
| KN1 | KN1F  KN1R | 5'-ATTGGGATAATGATGGTTTG-3'  5'-ATAGGAACAGCCCAGTAAAA-3' | 890 bp | AB924614 | This study |
| KN2 | KN2F  KN2R | 5'-TGTCCTTAGTCACAAATAAGTC-3'  5'-CTCTTTGGTATATAGCTGTGAA-3' | 1004 bp | AB371290 | This study |
| *Wzi* | wzi | 5′-GTGCCGCGAGCGCTTTCTATCTTGGTATTCC-3′  5′-GAGAGCCACTGGTTCCAGAA[C or T]TT[C or G]ACCGC-3′ | 580 bp |  | (27) |
| *Wza* |  | 5′-TGAAAGTGTTTGTCATGGG-3′  5′-TTCAGCTGGATTTGGTGG-3′  5′-GCTTCCATCATTGCAAAATG-3′ |  |  | (28) |
| *Wzc* |  | 5′-GGGTTTTTATCGGGTTGTAC-3′  5′-TTCAGCTGGATTTGGTGG-3′  5′-GCTTCCATCATTGCAAAATG-3' |  |  | (28) |

Supplementary Table 2. Virulence-associated genes detected in this study and performed as previously published (7)

| Target gene | Primer name | Primer used | Expected amplicon (bp) |
| --- | --- | --- | --- |
| *clbA* | ClbA 1F | ATGAGGATTGATATATTAATTGGACA | 735 |
|  | ClbA 1R | TCAATTCTGCCCATTTGACG |  |
| *entB* | entB-F | ATTTCCTCAACTTCTGGGGC | 371 |
|  | entB-R | AGCATCGGTGGCGGTGGTCA |  |
| *iroN* | iroN-F | GTCCGGCGGTAACTTCAGCC | 829 |
|  | iroN-R | TCAGAATGAAACTACCGCCC |  |
| *iucA* | iucA-F | ATAAGGCAGGCAATCCAG | 2927 |
|  | iucA-R | TAACGGCGATAAACCTCG |  |
| *iutA* | iutA-F | GGCTGGACATCATGGGAACTGG | 300 |
|  | iutA-R | CGTCGGGAACGGGTAGAATCG |  |
| *rmpA* | rmpA-F | TACATATGAAGGAGTAGTTAAT | 505 |
|  | rmpA-R | GAGCCATCTTTCATCAAC |  |
| *rmpA2* | rmpA2-F | TGTGCAATAAGGATGTTACATTAGT | 609 |
| *rmpA2* | rmpA2-R | TTTGATGTGCACCATTTTTCA |  |
